# Premature aging of the hippocampal neurogenic niche in adult *Bmal1*‐ deficient mice

**DOI:** 10.18632/aging.100764

**Published:** 2015-06-27

**Authors:** Amira A. H. Ali, Beryl Schwarz‐Herzke, Anna Stahr, Timour Prozorovski, Orhan Aktas, Charlotte von Gall

**Affiliations:** ^1^ Institute for Anatomy II, Medical Faculty, Heinrich Heine University, D‐40225, Düsseldorf, Germany; ^2^ Department of Neurology, Medical Faculty, Heinrich Heine University, D‐40225 Düsseldorf, Germany

**Keywords:** adult neurogenesis, clock genes, dentate gyrus, bromodeoxyuridine, oxidative stress, Sirt1, PMP70, circadian disruption

## Abstract

Hippocampal neurogenesis undergoes dramatic age‐related changes. Mice with targeted deletion of the clock gene *Bmal1* (Bmal1^‐/‐^) show disrupted regulation of reactive oxygen species homeostasis, accelerated aging, neurodegeneration and cognitive deficits. As proliferation of neuronal progenitor/precursor cells (NPCs) is enhanced in young Bmal1^‐/‐^ mice, we tested the hypothesis that this results in premature aging of hippocampal neurogenic niche in adult Bmal1^‐/‐^ mice as compared to wildtype littermates. We found significantly reduced pool of hippocampal NPCs, scattered distribution, enhanced survival of NPCs and an increased differentiation of NPCs into the astroglial lineage at the expense of the neuronal lineage. Immunoreaction of the redox sensitive histone deacetylase Sirtuine 1, peroxisomal membrane protein at 70kDa and expression of the cell cycle inhibitor *p21* Waf1/CIP1 were increased in adult Bmal1^‐/‐^ mice. In conclusion, genetic disruption of the molecular clockwork leads to accelerated age‐dependent decline in adult neurogenesis presumably as a consequence of oxidative stress.

## INTRODUCTION

Generation of functional neurons from precursors is not restricted to prenatal and early postnatal development, but occurs also in the adult brain [1-3]. Adult neurogenesis can be found in two mammalian brain regions: the subgranular zone (SGZ) of hippocampal dentate gyrus (DG) [4] and the (subventricular zone) SVZ of the lateral ventricle [5]. In SGZ, adult neurogenesis is a multistep process which includes: (1) proliferation of neural stem/progenitor cells (NPCs), (2) migration and differentiation, (3) death or survival of the newly-generated cells (4) and, finally, maturation of the latter by acquiring the morphological and electro-physiological properties of mature granular neurons and their integration within the hippocampal synaptic network [2].

Several studies have indicated that adult neurogenesis in the SGZ is essential for regular hippocampal functions such as learning, memory formation and cognition [6-8] as well as stress resilience [9]. Importantly, adult neurogenesis is highly dynamic and modulated by various internal and external factors, including signaling within the local stem cell niche [10], aging [11], brain disorders [12], stressful experience [9], sleep deprivation [13] and circadian molecular clock [14–17].

Circadian clocks provide an internal time keeping system to coordinate physiology, metabolism and behaviour with changes in the environment within the 24 hour solar day [18, 19]. On the molecular level, the circadian clockwork is composed of two interlocked transcription/translation feedback loops (TTFL) generating rhythmic gene expression of approximately 24hour. The transcriptional activators brain and muscle Arnt-like protein1 (BMAL1) and circadian locomotor output cycles (CLOCK) heterodimerize, bind to E-box within the genes’ promoter and enhance the transcription of clock genes such as Period (*Per*) and Cryptochrome (*Cry*) as well as clock controlled genes. PER and CRY proteins translocate into the nucleus, heterodimerize and inhibit CLOCK:BMAL1 mediated transcription and consequently their own expression. In addition, CLOCK:BMAL1 complex activates transcription of nuclear receptors, REV-ERBa and RORa which regulate *Bmal1* transcription [20, 21].

Mice with a targeted deletion of the core clock gene *Bmal1* (Bmal1^−/−^) show high levels of reactive oxygen species (ROS) in different organs including the brain, cardinal symptoms of premature aging as well as impairment in learning and memory formation at the age of 16-18 weeks [22, 23]. This phenotype is probably a consequence of increased activity of mammalian target of rapamycin complex 1 (mTORC1), which is known to be associated with accelerated aging [24]. Moreover, Bmal1^−/−^ mice show changed cellular redox homeostasis and various symptoms of neuro-degeneration [25]. Further age-dependent attenuation of *Bmal1* expression was observed in selective brain regions, including the hippocampus [26]. These data suggest the importance of functional clockwork for the timing of (brain) aging.

Recent studies showed increasing evidence that the circadian molecular clock modulates NPC proliferation. Time-of-day dependent changes in NPC proliferation are abolished in mPer2- and Bmal1-deficient mice [17]. Moreover, NPC proliferation is increased in mPer2- [14, 17], Rev-erbα - [16] or young (5 weeks old) Bmal1-deficient mice [17], suggesting a link between the molecular clockwork and the NPC cell cycle. However, the proliferation rate in hepatocytes of adult Bmal1^−/−^ mice is decreased as a consequence of down-regulation of the regulator of cell cycle progression *wee1* [27] and up-regulation of the cell cycle inhibitor *p21^WAF1/CIP1^* [28], suggesting accelerated senescence.

In addition, it has been shown that peroxisomes are involved ROS homeostasis and play a critical role in regulating cellular aging [29] including physiological and accelerated brain aging [30]; furthermore, the redox sensitive protein deacetylase Sirtuine 1 (Sirt1) is tightly linked to the circadian clock [31-33] and is involved in aging and cellular senescence [34]. Thus, peroxisomal function and Sirt1 could be a possible link between the molecular clockwork, ROS homeostasis and the timing of aging or cellular senescence.

Therefore, we tested the hypothesis that adult Bmal1-deficient mice show accelerated aging within the hippocampal neurogenic niche as a consequence of oxidative stress. We systematically analyzed neurogenesis in the SGZ including proliferation, differentiation and survival of NPCs in adult Bmal1^−/−^ mice. Moreover, peroxisomal membrane protein of 70 kDa (PMP70), Sirt1-immunoreaction as well as the expression of genes involved in ROS homeostasis: peroxiredoxin 1 (*prdx1)* and metallothionein (*mt*) [35-37], cell cycle regulation: *p21* ^Waf1/CIP1^, *cyclin D1* (*cd1*) and *wee1* [28] and genes encoding for neurotrophic factors (*fgf* and *bdnf*) [38, 39] were analyzed.

In Bmal1^−/−^ mice, we found a significantly reduced number of BrdU-immunopositive NPCs, a shifted differentiation of NPCs towards astroglial at the expense of neuronal lineage, higher immunoreaction of Sirt1 in addition to increased expression level of the cell cycle inhibitor *p21* ^Waf1/CIP1^. These findings suggest accelerated aging of the neurogenic niche in adult Bmal1^−/−^ mice presumably as a consequence of, oxidative stress, peroxisomal dysfunction and Sirt1 activation.

## RESULTS

### The pool of NPCs in DG was reduced in Bmal1^−/−^ mice

**Figure 1:**
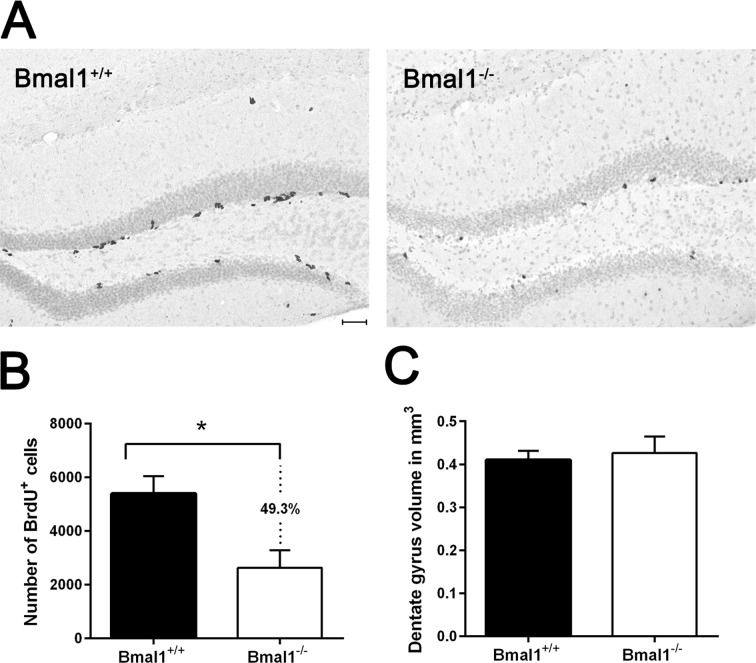
The pool of NPCs was reduced in DG in Bmal1‐/‐ mice BrdU‐labeled cells in DG were analyzed one day after the final BrdU administration. (**A**) Representative photomicrographs of BrdU‐positive (^+^) NPCs. (**B**) Quantification of BrdU‐immunoreaction in DG of one hemisphere. Total number of BrdU ^+^ cells was significantly reduced in Bmal1^‐/‐^ as compared to Bmal1^+/+^. (**C**) Estimation of total volume of DG didn’t show significant difference between both genotypes. Values are shown as mean + SEM, *: *P* < 0.05, scale bar = 50 μm.

To analyze whether phenotypical changes associated with premature aging in Bmal1^−/−^ mice [23] are associated with alteration in hippocampal neurogenesis, we used adult male mice 10–15 weeks old, just before the onset of growth retardation. One day after the final bromodeoxyuridine (BrdU) administration, BrdU staining confirmed the presence of proliferating NPCs in the SGZ and GCL in hippocampus of both genotypes. BrdU-positive cells exhibited homogenously stained triangular, elongated or rounded nuclei. In SGZ, BrdU-positive cells were arranged in clusters, whereas in GCL BrdU-positive cells were distributed sporadically (Fig. 1A). In Bmal1^−/−^ mice, the number of BrdU-positive cells (2805±743.5) was significantly reduced (by about 49.3%) as compared to Bmal1^+/+^ mice (5537±605.8) (*P*=0.026) (Fig.1B), indicating that total NPC pool was reduced in Bmal1^−/−^ mice.

To determine if the decline in neurogenesis is associated with reduction in the total DG volume, Cavalieri volume estimation [40] was performed. There was no significant difference in the DG volume between Bmal1^−/−^ (0.42 ± 0.04mm^3^) and Bmal1^+/+^ mice (0.41 ± 0.02 mm^3^) (*P*=0.72) (Fig.1C).

### The spatial distribution of NPCs in DG was changed in Bmal1^−/−^ mice

**Figure 2:**
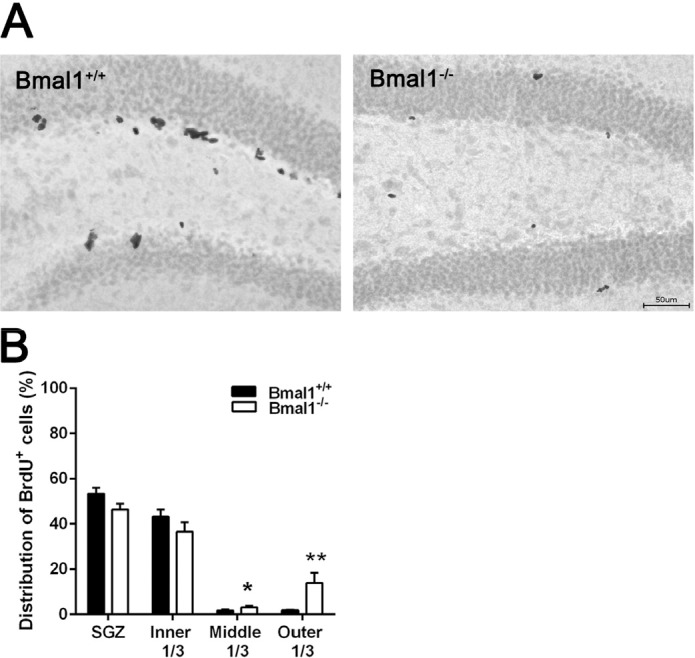
The spatial distribution of NPCs in DG was changed in Bmal1‐/‐ mice (**A**) Representative photo‐ micrograph of distribution of BrdU^+^ NPCs one day after the last BrdU injection. (**B**) In Bmal1^‐/‐^ a larger fraction of NPCs is located in middle and outer thirds

In both genotypes, the majority of BrdU-positive cells were located in SGZ (Bmal1^+/+^: 53.3±2.7%; Bmal1^−/−^: 46.4±2.5%) and inner third of GCL (Bmal1^+/+^: 43.2±3.1%; Bmal1^−/−^: 36.6±4.2%). In Bmal1^−/−^ mice, a significantly larger fraction of BrdU-positive cells was located in the middle third (Bmal1^+/+^: 1.7±0.5%; Bmal1^−/−^: 3.1±0.5%) (*P*=0.026) and the outer third (Bmal1^+/+^: 1.7±0.3%; Bmal1^−/−^: 13.9±4.5%) (*P*=0.004) as compared to Bmal1^+/+^ mice (Fig. 2A, B).

### The pool of DCX-positive progenitors and the total number of developing neurons were reduced in Bmal1^−/−^ mice

**Figure 3:**
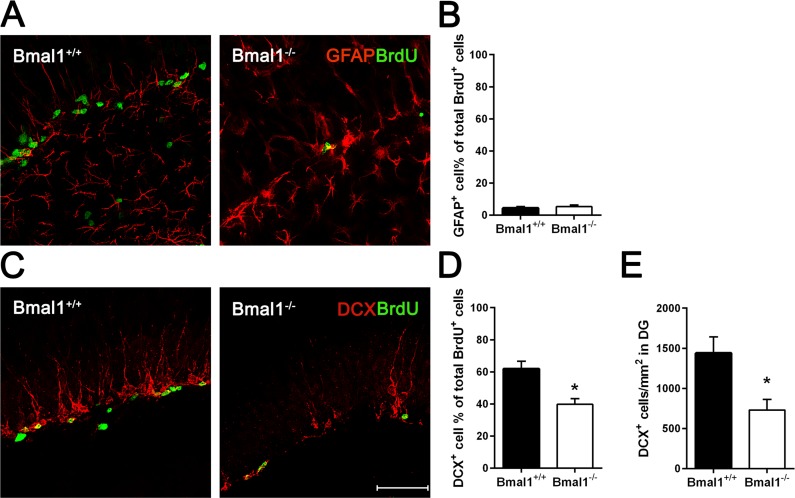
The pool of DCX‐positive progenitors and the total number of developing neurons were reduced in Bmal1‐/‐ mice Double labeling of BrdU and GFAP or DCX in DG was analyzed one day after the final BrdU administration. (**A**) Representative photomicrographs showing cells co‐labeled for BrdU (green) and GFAP (red). (**B**) Quantification of the percentage of GFAP^+^ cells among all BrdU^+^ cells. In both genotypes, the percentage of cells co‐labelled for GFAP and BrdU was equally low. (**C)** Representative photomicrographs showing cells co‐labeled for BrdU (green) and DCX (red). (**D**) Quantification of the percentage of DCX^+^ cells among all BrdU^+^ cells. The percentage of total BrdU^+^ cells expressing DCX was significantly reduced in Bmal1^‐/‐^. (**E**) The total number of DCX^+^ cells per mm^2^ in DG was significantly reduced in Bmal1^‐/‐^. Values are represented as mean + SEM, *: *P* < 0.05, scale bar = 50μm.

In order to differentiate precursor subtypes, BrdU was co-labelled with GFAP (for detection of early neural progenitors) or DCX (for detection of type2b and type 3 progenitors) [41]. In both genotypes, only a small fraction of BrdU positive cells were co-labelled with GFAP. We found no significant difference in the percentage of BrdU/GFAP-co-labeled early neural progenitors between Bmal1^−/−^ mice (5.3±1.04%) and Bmal1^+/+^ mice (4.4±0.96%) (*P*=0.699) (Fig. 3A, B), suggesting that Bmal1-deficiency did not affect the proliferation of early neural progenitors. However, the percentage of cells co-labeled with BrdU and DCX was significantly larger in Bmal1^+/+^ mice (61.9±4.6%) as compared to Bmal1^−/−^ mice (39.7±3.6%) (*P*=0.015) (Fig. 3C, D). Moreover, the total number of DCX positive cells/developing neurons was significantly reduced in Bmal1^−/−^ mice (729.8 ± 133.6) as compared to Bmal1^+/+^ mice (1440 ± 202.3) (*P*=0.032) (Fig. 3E). These findings denote that specifically the pool of DCX-positive type 2b and type 3 progenitors was affected in Bmal1^−/−^ mice.

### Survival of NPCs was enhanced in Bmal1^−/−^ mice

**Figure 4:**
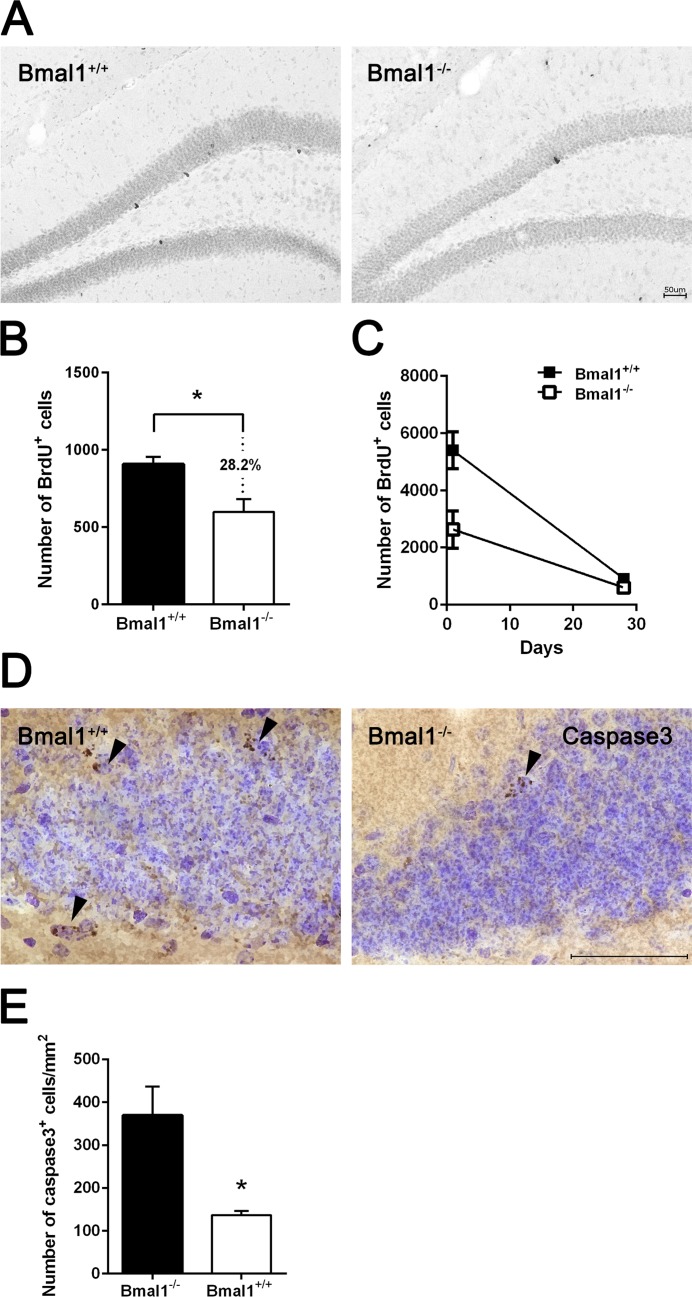
Survival of NPCs was enhanced in Bmal1‐/‐ mice Surviving BrdU^+^ cells were analyzed 28 days after the final BrdU administration. (**A**) Representative photomicrographs of BrdU^+^ cells. (**B**) Quantification of BrdU+ cells in DG. The number of BrdU+ cells was significantly smaller in Bmal1^‐/‐^ as compared to Bmal1^+/+^. (**C**) Linear regression of BrdU^+^ cells one day and 28 days after final BrdU‐injection showed significantly steeper slope in Bmal1^+/+^ as compared to Bmal1^‐/‐^. (**D**) Representative photomicrographs showing caspase3^+^ cells. (**E**) Quantification of caspase3^+^ cells in DG. The number of caspase3^+^ cells was significantly lower in Bmal1^‐/‐^ as compared to Bmal1^+/+^. Values are given as mean + SEM, *: *P* <0.05, scale bar = 50 μm.

Survival of NPCs in DG was analyzed 28 days after the last BrdU administration. The number of BrdU positive cells was significantly reduced by about 28.2% in Bmal1^−/−^ mice (647±83.9) as compared to Bmal1^+/+^ mice (901.2±52.97) (*P*=0.03) (Fig. 4A, B), consistently with a reduced total number of NPCs in adult Bmal1^−/−^ mice. Linear regression revealed a steeper slope in Bmal1^+/+^ mice as compared to Bmal1^−/−^ mice (*P*=0.028) (Fig. 4C), indicating a higher survival rate in Bmal1^−/−^ mice. Consistently, the number of caspase3^+^ cells/mm2 in DG was significantly decreased in Bmal1^−/−^ (136.5±5.1) as compared to Bmal1^+/+^ mice (369.3 ± 33.60) (*P*=0.028) (Fig. 4D, E), indicating decreased cell death in DG of Bmal1^−/−^ mice.

### Fate decision of NPCs was altered in Bmal1^−/−^ mice

**Figure 5:**
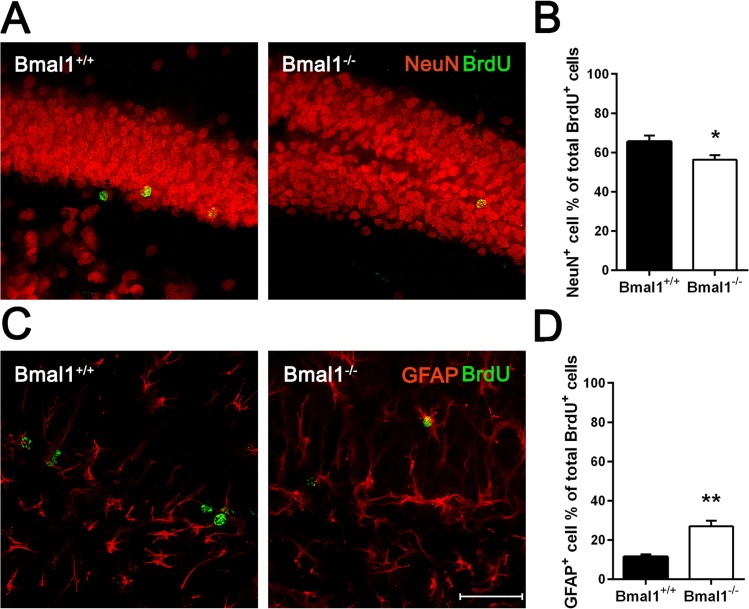
Fate decision of NPCs was altered in Bmal1‐/‐ mice Double labeling of BrdU with the marker for adult neurons NeuN and with the marker for astrocytes GFAP was analyzed 28 days after the final BrdU administration. (**A**) Representative photomicrographs of double labeling for BrdU (green) and NeuN (red). (**B**) Quantification of the percentage of NeuN^+^ cells among all BrdU^+^ cells. The percentage of BrdU/NeuN double labeled cells was significantly smaller in Bmal1^‐/‐^. (**C**) Representative photomicrographs of double labeling for BrdU (green) and GFAP (red). (**D**) Quantification of the percentage of GFAP^+^ cells among all BrdU^+^ cells. The percentage of BrdU/ GFAP double labeled cells was significantly higher in Bmal1^‐/‐^. Values are given as mean + SEM,*: *P* <0.05, ^**^: *P* <0.01, scale bar = 50μm.

Differentiation of NPCs was assessed 28 days after the last BrdU administration. Neuronal differentiation was analyzed by calculating the percentage of cells co-expressing BrdU and neuronal marker NeuN. The percentage of BrdU/NeuN positive cells was significantly lower in Bmal1^−/−^ mice (56.31± 2.24%) as compared to Bmal1^+/+^ mice (65.48± 3.11%) (*P*=0.033) (Fig. 5A, B). In contrast, the percentage of cells co-expressing BrdU and the astrocyte marker GFAP was significantly higher in Bmal1^−/−^ mice (26.96± 2.81%) as compared to Bmal1^+/+^ mice (11.45±1.128%) (*P*=0.008) (Fig. 5C, D). Based on these findings, we conclude that the fate decision of neuronal progenitors was shifted towards the astroglial lineage at the expense of the neuronal lineage in Bmal1^−/−^ mice.

### PMP70-expression was increased in Bmal1^−/−^ mice

**Figure 6:**
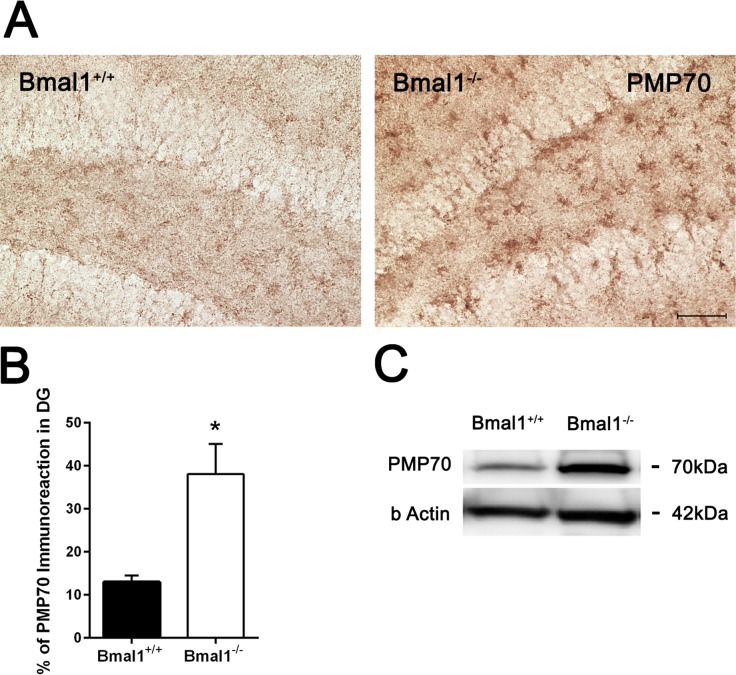
PMP70 expression was increased in Bmal1‐/‐ mice (**A**) Representative photomicrographs of PMP70‐ immunoreaction in DG. (**B**) Quantification of PMP70 immunopositive area in DG. Immunoreaction was significantly higher in Bmal1^‐/‐^. (**C**) Representative immunoblot of PMP70 in hippocampal protein extract. Stronger immunopositive bands were observed in Bmal1^‐/‐^. Values are shown as mean + SEM, *: *P* <0.05, scale bar = 50 μm.

As peroxisomes play an important role in ROS homeostasis and cellular aging, expression of peroxisome-related protein PMP70 was analyzed. Immunohistochemistry and immunoblotting revealed higher PMP70 levels in the DG of Bmal1^−/−^ mice as compared to Bmal1^+/+^ mice (*P*=0.024) (Fig. 6).

### Sirt1-immunoreaction was increased in Bmal1^−/−^

**Figure 7:**
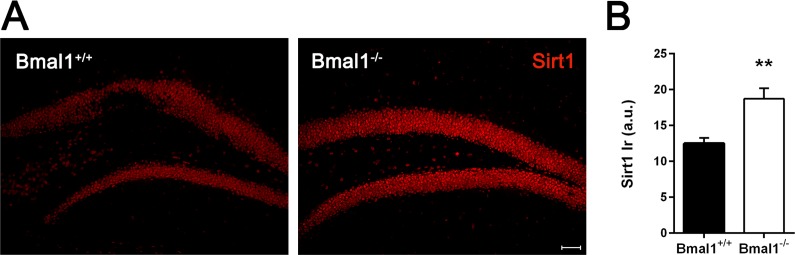
Sirt1‐immunoreaction was increased in Bmal1‐/‐ mice **(A)** Representative photomicrographs of Sirt1‐immunoreaction in DG **(B)** Quantification of Sirt1 immunoreaction (Ir). Sirt1‐Ir was significantly higher in Bmal1^‐/‐^. Values are shown as mean + SEM, ^**^: *P* <0.01, scale bar = 50μm.

Considering associated pathways responsible for disturbed proliferation and differentiation of neural progenitors, we assessed the possible involvement of Sirt1. This NAD+-dependent histone deacetylase (HDAC) has been shown to play a crucial role in regulating redox-dependent fate of neural progenitors [33] and to interact with circadian promoters in a cyclic manner [42]. Indeed, the intensity of Sirt1-immunoreaction in the DG of Bmal1−/− mice was significantly higher as compared to Bmal1+/+ mice (*P*=0.004) (Fig. 7).

### Age-dependent changes in gene expression levels in Bmal1^−/−^ mice

**Figure 8:**
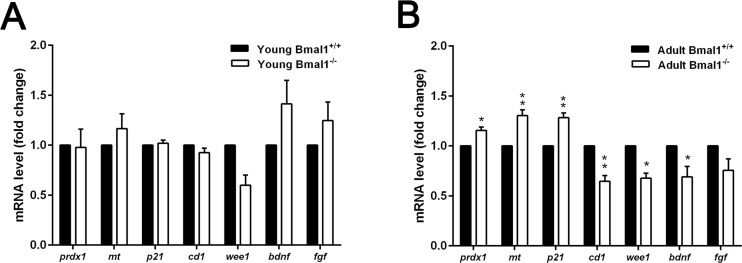
Age‐dependent changes in gene expression in Bmal1‐/‐ mice (**A**) Expression of genes remained stable in young mice (6 weeks old) and didn’t show significant difference between both genotypes. (**B**) Expression levels of ROS‐induced genes *prdx 1* and *mt* as well as cell cycle inhibitory gene *p21*^Waf1/CIP1^ *(p21)* were significantly higher, while *cd1*, *wee1*and *bdnf* were down‐regulated in hippocampus of adult Bmal1**^‐/‐^** (12 weeks old). Expression level of *fgf* was not different between both genotypes. Values are shown as mean + SEM. *: *P* < 0.05, ^**^: *P* < 0.01.

Further analysis of genes induced by oxidative stress (*prdx1, mt*), cell cycle genes (*p21^Waf1/CIP1^, cd1, wee1*) in addition to genes that encode for neurotrophic factors (*bdnf, fgf*) was performed. We compared the expression of these genes between Bmal1^+/+^ and Bmal1^−/−^ mice of two different ages: young mice (6 weeks old) and adult mice (12 weeks old). In adult mice, ROS sensitive genes *prdx1* and *mt* were significantly up-regulated in Bmal1^−/−^ (*P*=0.0142, *P*=0.00612; respectively) as compared to Bmal1^+/+^ mice, consistently with high oxidative stress in the brain of Bmal1^−/−^ mice [22]. In addition, in Bmal1^−/−^ mice, expression level of *p21^Waf1/CIP1^* was significantly higher, whereas expression levels of *cd1* and *wee1* (*P*=0.003) were significantly lower as compared to Bmal1^+/+^ mice. Expression of *bdnf* was down regulated in Bmal1^−/−^ mice (*P*=0.04). In contrast to this, there were no significant differences in expression levels between both genotypes in young mice. Expression of *fgf* was not different between the two genotypes in both ages (Fig. 8).

## DISCUSSION

The present study demonstrated novel influence of genetic disruption of the molecular clockwork on adult neurogenesis and thus neuronal plasticity. In comparison to their wildtype littermates, Bmal1^−/−^ mice showed a reduced pool of hippocampal NPCs, a scattered distribution of NPCs and an increased differentiation of NPCs into the astroglial lineage at the expense of the neuronal lineage, reminiscent of the regular aging process in the hippocampal neurogenic niche. Moreover, ROS-sensitive genes, the peroxisomal protein PMP70 and the redox sensitive histone deacetylase Sirt1showed higher levels in Bmal1^−/−^ mice. Finally, expression levels of genes encoding for regulators of cell cycle control were significantly altered in Bmal1^−/−^ mice; indicating that high oxidative stress, as a consequence of circadian disruption, leads to premature aging of the neurogenic niche.

**Figure 9:**
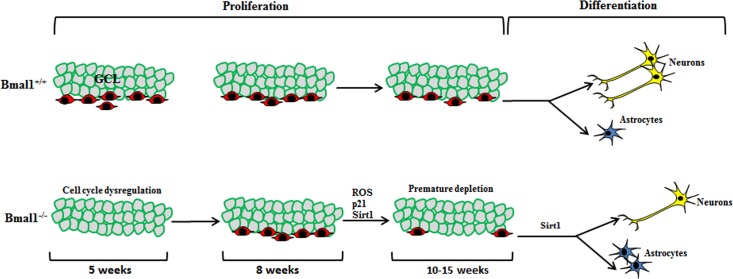
Schematic illustration summarizing the observed differences in neurogenesis in DG between Bmal1‐/‐ and Bmal1+/+ In young (5 weeks old) Bmal1^‐/‐^ mice there was an enhanced proliferation of hippocampal NPCs probably due to dysregulated cell cycle [17]. In young adult (8 weeks old) Bmal1^‐/‐^ mice, no difference in proliferating hippocampal NPCs was found [15], indicating that the promoting effect of Bmal1‐deficiency on NPC mitotic activity is transient and age‐dependent. In our study, adult Bmal1^‐/‐^ (10‐15 weeks old) mice showed a significant decrease in the pool of hippocampal NPCs indicating a dramatic down‐regulation in proliferation activity of NPCs presumably due to ROS‐induced accelerated aging/senescence. Moreover, NPCs of adult Bmal1^‐/‐^ mice showed a shifted differentiation towards the astroglial lineage at the expense of neurons, presumably as a consequence of Sirt1 activation.

An earlier study [17] in young (5 weeks old) Bmal1^−/−^ mice showed enhanced proliferation of hippocampal NPCs. Using mathematical models, they postulated that dysregulated clock-driven expression of a cell cycle inhibitor targets is attributed to enhanced entry and diminished cell cycle exit in Bmal1^−/−^ progenitors. In contrast to 5 weeks old mice, no difference in proliferating capacity of hippocampal NPCs was found in 8 weeks old Bmal1^−/−^ mice [15], indicating that the promoting effect of Bmal1-deficiency on mitotic activity is transient and age-dependent. In our study, we used adult mice (10-15 weeks old); this age is of particular interest, as it is before the onset of systemic growth retardation as a hallmark of accelerated aging in 16-18 weeks old Bmal1^−/−^ mice [23], and before the development of neuropathologies in 24 weeks old Bmal1^−/−^ mice [25]. We observed a significant decrease in the pool of hippocampal NPCs in Bmal1^−/−^ as compared to Bmal1^+/+^ littermates. This indicates that, at the age between 5 and 10 weeks, there was a dramatic down-regulation in proliferation activity of NPCs presumably due to accelerated aging/senescence in Bmal1^−/−^ mice (Fig. 9).

Generally, the number of hippocampal NPCs markedly decreases between young (3 weeks old) and young adult mice (8 weeks) and also between young adult and adult mice (20 weeks). This age-related decrease in hippocampal neurogenesis is probably due to a depletion of the stem cell pool, as a consequence of a rapid succession of asymmetric cell divisions, giving rise to a decreasing amount of new neurons and an increasing amount of mature astrocytes [43]. Thus, the initial enhanced proliferation in young Bmal1^−/−^ [17] may be responsible for premature “division-coupled depletion” of NSCs in adult Bmal1^−/−^ mice. This is reminiscent of FoxO-deficient mice, which also show initial enhanced proliferation of NPCs followed by premature exhaustion of NSCs pool and reduction in neurogenesis [44, 45]. Moreover, our findings are in agreement with studies in mice with a targeted deletion of the negative regulator of Bmal1, Rev-erbα, showing enhanced hippocampal adult neurogenesis [16]. Moreover, in adult Bmal1^−/−^ mice, we found a significantly larger fraction of NPCs in middle and outer thirds of GCL, which is consistent with an age-related change in the spatial distribution of new-born neurons observed previously [46].

Based on the characterization of BrdU-positive precursor cells described before [41], we analyzed precursor cell subtypes. Interestingly, we did not find a significant decrease of GFAP/BrdU co-labelled type 1 radial-glia like cells but a significant reduction of the pool of DCX/BrdU co-labelled type 2b and type 3 cells. This is consistent with most prominent decline in type 2b and type 3 precursor cells from adult to old (16 month) mice [46] and is considered as a reliable feature of impaired neurogenesis [47]. Therefore, we postulated that Bmal1-deficiency specifically affected the pool of type 2b and type 3 cells.

Adult Bmal1^−/−^ mice show a higher survival rate of NPCs in the DG as compared to Bmal1^+/+^ mice. This is consistent with the previously observed enhanced survival in young adult Bmal1^−/−^ mice interpreted as impaired pruning of newly generated neurons [15]. Moreover, adult Bmal1^−/−^ mice showed a significantly lower number of apoptotic cells with no change in DG volume consistent with the decline in cell death during aging in combination with a stable DG volume from 6 weeks of age onwards [40]. Differentiation of NPCs was analyzed 28 days after the last BrdU administration. We found a significantly lower percentage of BrdU/NeuN-positive cells in Bmal1^−/−^ as compared to Bmal1^+/+^ mice. This is consistent with the reduced total amount of DCX-positive cells and suggests an impaired neuronal differentiation. In parallel, we found a significantly higher percentage of BrdU/GFAP-positive cells in Bmal1^−/−^ as compared to Bmal1^+/+^ mice, suggesting a possible shift towards an astroglial lineage commitment, consistently with the disposable stem cell model proposed by Encinas and colleagues [43]. Importantly, 10 weeks old Bmal1^−/−^ mice already show astrogliosis [25], which may be indicative for stress-induced activation of astrocytes.

In Bmal1^−/−^ mice, ROS homeostasis is affected resulting in significantly higher ROS level in the peripheral tissues [48] as well as brain extracts [22], indicating a role of BMAL1 in neuronal redox homeostasis. Besides, Bmal1^−/−^ mice showed a disrupted circadian oscillation of intracellular ROS level and increased sensitivity to oxidative stress leading to stress-induced senescence [48]. High level of ROS in the brain of Bmal1^−/−^ mice coincides with impaired cognitive performance and behavioural abnormalities as well as astrogliosis, neuropathology, neurodegeneration and expression of genes encoding for proteins involved in oxidative stress and redox defence mechanisms [22, 25]. Oxidative stress leads to decreased proliferation and neuronal differentiation capacity, and induces cellular senescence in NPCs [33, 49]. It also activates compensatory mechanisms to minimize cell death by interference with apoptotic signalling pathways [49]; which is consistent with enhanced survival of NPCs shown in this study and observed previously in Bmal1^−/−^ mice [15].

Peroxisomes play a critical role in production and elimination of ROS. Low concentration of peroxisomal ROS stimulates anti-aging mechanism, while higher concentrations trigger pro-aging pathways [29]. Adult Bmal1^−/−^ mice showed an increased expression of peroxisomal marker PMP70. This in consistent with the age-dependent increase in PMP70 expression which correlates with gliosis [30], and with increased expression of ROS-sensitive genes prdx1 and mt. Importantly, only adult but not young Bmal1^−/−^ mice showed significant higher expression levels of *prdx1* and *mt*, indicating an age-dependent increase in oxidative stress in Bmal1^−/−^ mice.

The molecular clockwork is tightly linked to the ROS-sensitive histone deacetlylase Sirt1 [31, 32, 50-55]. Sirt1 controls *Bmal1* and *Clock* transcription [32], affects CLOCK/BMAL1-mediated gene expression [51, 53] and modulates PER2 stability [31]. Within the circadian rhythm generator, the suprachiasmatic nucleus, Sirt1 is higher in young animals as compared to old animals and might play a role in circadian rhythm stability which declines with age [32]. Sirt1 is activated by low-level DNA damage stimuli such as mild oxidative stress; it modulates proliferation, cell cycle arrest, differentiation and promotes cellular senescence [56–59]. Brain specific deletion of Sirt1 is associated with enhanced proliferation of hippocampal NPCs, suggesting a role of Sirt1 in control of self-renewal [59]. In NPCs, mild oxidative stress leads to the activation of Sirt1 with subsequently suppression of neurogenesis and increase of astroglial differentiation at the expense of neuronal differentiation [33]. We found a significant higher Sirt1-immunoreaction in the DG of adult Bmal1^−/−^ as compared to their Bmal1^+/+^ littermates. Taken together, our observations are in line with an activation of Sirt1 by oxidative stress, its negative effect on NPCs proliferation and neuronal differentiation, and its promoting effect on astrogenesis [33, 59].

The cell cycle inhibitor *p21*^Waf1/CIP1^ is considered to be an important link between the molecular clockwork and the cell cycle [28]. *P21*^Waf1/CIP1^ is a target of *p53* which is induced during replicative senescence in response to chronic sublethal genotoxic stress by Sirt1 [57]. Furthermore, *p21* ^Waf1/CIP1^ was reported to be negative regulator of NSCs proliferation through oxidative stress mediated *p38* MAPK signaling [60]. Moreover, *p21*^Waf1/CIP1^ has been reported to play a key role during stem cell aging. Accumulation of DNA damage, associated with aging, induces *p21 ^Waf1/CIP1^* - dependent check point leading to cell cycle arrest with subsequent depletion of stem/progenitor cells, impaired organ maintenance and reduced life span [61]. Also in adipocytes, oxidative stress leads to accelerated aging by induction of *p21 ^Waf1/CIP1^* [62]. Bouchard-Cannon et al. proposed higher level of *p21 ^Waf1/CIP1^*, leading to cell cycle dysregulation and enhanced proliferation in young (5 weeks old) Bmal1^−/−^. This is in contrast to our observation of similar *p21*^Waf1/CIP1^ gene expression levels in young (6 weeks old) Bmal1^−/−^ and Bmal1^+/+^ mice. However, we found a significant increase in *p21*^Waf1/CIP1^ gene expression levels in adult (12 weeks old) Bmal1^−/−^ mice. This was paralleled by a significant decrease in the expression levels of *cd1* which regulates the activity of the cyclin-dependent-kinase complex whose activity is required for cell cycle G1/S transition, and of the clock controlled gene *wee1* which regulates the G_2_/M transition [27]. These finding are in agreement with the up-regulation of *p21*^Waf1/CIP1^ and the down-regulation of *wee1* in adult Bmal1^−/−^ hepatocytes, exhibiting a decreased proliferation rate [28]. Our findings suggest premature depletion of neuronal progenitor cells in adult Bmal1^−/−^ mice by the induction of *p21 ^Waf1/CIP1^* expression.

The neurotrophic factors BDNF and FGF play an important role in NPCs proliferation, differentiation, survival and function, and mediate neuronal plasticity and cognitive function [63]. Expression levels of genes encoding for neurotrophic factors do not change with age [64]. However, oxidative stress has been shown to affect *a bdnf* expression level [65], which is consistent with our findings of reduced expression level of *bdnf* in adult Bmal1^−/−^ mice.

In summary, our data indicate that accumulation of ROS in adult Bmal1^−/−^ leads to an increased cell-cycle arrest of neuronal precursors cells which interferes with the initially enhanced proliferation of neuronal stem cells observed in young Bmal1^−/−^ mice [17]. The dramatic decrease in proliferation and neuronal differentiation capacity in Bmal1^−/−^ mice within the time range of 6 weeks is probably a result of both: premature aging due to division-coupled depletion and ROS-stress-induced cellular senescence. Therefore, genetic interference with the core molecular clockwork affects adult neurogenesis and thus neuronal plasticity. In this context, it is highly relevant to further study the effects of circadian disruption in general on adult neurogenesis, neuronal plasticity and brain aging.

## MATERIALS AND METHODS

### Animals

All animal procedures were approved by the local government, North Rhine-Westphalia State Agency for Nature, Environment and Consumer Protection, Germany (AZ: 84-02.04.2012.A102, 84-02.04.2014.A314) and conform to international guidelines on the ethical use of animals [66].

Mice heterozygous for a targeted deletion of Bmal1 (Bmal1^+/−^) were kindly provided by Christopher Bradfield [67]. Mice were kept for breeding at the local animal facility (University of Düsseldorf). Bmal1^−/−^ and Bmal1^+/+^ were obtained by breading of Bmal1^+/−^ male with Bmal1^+/−^ female. Genotype was confirmed by PCR [67].

Only male adult (10-15 weeks old) and young (6 weeks old) mice were used. Mice were housed in standard cages and had free access to food and water in a temperature controlled environment with 12 h light and 12 h darkness, zeitgeber time (ZT) 0 referred to lights on at 6:00 am.

### BrdU administration

Each animal received an intraperitoneal injection of 100 mg/kg body weight of the S-phase marker BrdU (Roche, Switzerland) twice daily, at the beginning and the end of the light phase (ZT2 and ZT12, respectively) on three consecutive days. For analysis of NPCs, one group of mice (group 1, n= 6 of each genotype) was sacrificed 18 h (ZT6) after the last BrdU administration. For analysis of NPC survival and neuronal differentiation, a second group (group 2, n=6 of each genotype) was sacrificed 28 days (ZT6) after the last injection.

### Tissue processing

Mice were deeply anaesthetized using Ketamine/Xylazine (100mg/10mg respectively /kg body weight). Animals were perfused transcardially with 0.9% NaCl followed by 4% paraformaldehyde using Ministar Peristaltic Pump (World Precision Instruments, USA). Brains were removed from the skull, post fixed in 4% paraformaldehyde for 24 hours followed by cryoprotection in 20% sucrose for another 24 hours. Brains were sectioned through the entire rostro-caudal extent of the hippocampus into 40μm free floating coronal sections using a cryomicrotome (Reichert-Jung).

### Immunohistochemistry

Of each brain (group 1 and 2), every sixth section was subjected to immuno-histochemistry for BrdU with DAB as the chromogen in one reaction. All sections were permeabilized by phosphate buffered saline (PBS) with 0.2% Triton-X 100. Sections were incubated in 0.6% H_2_O_2_ for 30 minutes at room temperature, and then rinsed in PBS. DNA was denatured using 2N HCl for 30 minutes at 37°C followed by washing with 0.1 M boric acid for 10 minutes at room temperature. Sections were rinsed in PBS, incubated in 5% normal goat serum in PBS-T 0.2% for one hour at room temperature to block unspecific binding of secondary antibody. Sections were incubated with rat monoclonal anti BrdU antibody (1:800, AbD Serotec, UK) overnight at 4°C. After washing, sections were incubated with biotinylated goat anti rat IgG (1:500, Vector Laboratories, CA) for one hour at room temperature followed by incubation with VECTASTAIN^®^ Elite^®^ ABC solution (Vector Laboratories, CA) for one hour at room temperature. Sections were rinsed and incubated with 0.05% 3, 3′-Diaminobenzidine (SIGMA-ALDRICH, USA) for 4 minutes. Sections were rinsed, mounted on slides, dehydrated, counter-stained with Cresyl violet, and finally cover slipped using Depex (SERVA Electro-phoresis, Germany).

Every twelfth section was used for immuno-histochemical staining according to the above mentioned protocol without DNA denaturing step, using rabbit polyclonal anti PMP70 (1:2500, SIGMA-ALDRICH, USA) or rabbit monoclonal anti cleaved caspase3 (1:150, Cell Signaling, USA). Biotinylated goat anti rabbit IgG was used as secondary antibody (1:500, Vector Laboratories, CA).

### Immunofluorescence

From each brain of group 1, every twelfth section was used for double immunofluorescence with BrdU/DCX, BrdU/GFAP and for Sirt1 immunofluorescence. From each brain of group 2, every twelfth section was stained for BrdU/NeuN and BrdU/GFAP. For BrdU staining, DNA was denatured as described above. Sections were incubated with the following primary antibodies: rat monoclonal anti BrdU (1:500, AbD Serotec, UK) and rabbit polyclonal anti DCX (1:1000, Abcam, England); polyclonal rabbit anti GFAP (1:2000, DAKO, Denmark), polyclonal rabbit anti NeuN (1:1000, Millipore-Chemicon, CA) or polyclonal rabbit anti Sirtuine-1 (Sirt1, 1:1000, Millipore-Chemicon, CA). Sections were then rinsed in PBS, followed by incubation with the following secondary antibodies: Alexa Fluor 488 goat anti rat IgG (1:500, Molecular Probes, USA) and Alexa Fluor 568 goat anti rabbit IgG (1:500, Molecular Probes, USA) for one hour at room temperature. After washing, nuclei were counterstained with NucBlue Fixed Cell Stain (Molecular probes, USA). Sections were then mounted on slides and dehydrated. Finally slides were coverslipped using Vectashield Hard Set anti-fade reagent (Vector Laboratories, CA) and kept in darkness at 4° C.

### Protein extraction and western blotting

Adult male mice, from both genotypes, were killed by an overdose of isoflurane at the same time (ZT3) to avoid possible circadian variation of protein expression. Brains were removed quickly and snap frozen. Protein was extracted by homogenizing the hippocampus in lysis buffer (T-PER Protein Extraction Reagent, 1% Halt Protease Inhibitor Cocktail (Thermo scientific, USA)) using electric homogenizer. Homogenates were centrifuged at 16000 g at 4°C for 20 minutes, and then the supernatant was collected. Protein concentration was determined using Bradford protein assay [68]. Proteins were separated on 8% SDS-PAGE, and then transferred to Invitrolon^TM^ PVDF membrane (Life technologies, CA). Membrane was blocked using StartingBlock^TM^ (TBS) blocking buffer (Thermo scientific, USA), then incubated with rabbit polyclonal anti PMP70 (1:1000, SIGMA-ALDRICH, USA) or rabbit polyclonal anti actin (1:1000, SIGMA-ALDRICH, USA) overnight at 4°C. This was followed by washing then incubation with HRP-conjugated goat anti rabbit IgG one hour at room temperature. Immunopositive bands were detected using Immobilon^TM^ Western (Millipore, USA) and visualized by ChemiDoc^™^ MP System (Biorad).

### Image analysis

BrdU-immunoreactive cells (DAB) in the DG were counted manually using a 40X objective by bright field mode on a KEYENCE BZ 900E microscope (Japan). Cells were categorized according to their location in the SGZ and GCL of the DG. SGZ was defined as a two-nucleus-wide area along the inner border of the GCL towards the hilus, while the GCL was divided equally into an inner, middle and outer third [69]. As every sixth section was stained, the resulting cell number was multiplied by six to estimate the total number of BrdU-positive cells in the entire hippocampus.

Fluorescent signals were detected using fluorescence mode of KEYENCE BZ 900E (Japan) using a 40X objective and respective filters. Images were processed by BZ-II analyzer software (Keyence, Japan) by an observer blind to experimental condition. Settings (exposure time, photo interval, haze reduction condition) were kept identical during images acquisition and processing in all samples. Co-localization was confirmed by Z stack series and real-time 3D procedure. BrdU-positive cells were counted in the dorsal and ventral hippocampus of each animal. Fifty to sixty BrdU-positive cells per animal were analyzed for co-labeling of BrdU with GFAP or DCX. For detection of early neural progenitors, only GFAP positive cells in the SGZ and GCL that showed the morphological characters of radial glia-like cells [70] co-labeled with BrdU were counted. For analysis of neuronal or astroglial differentiation after 4 weeks, twenty to thirty BrdU-positive cells per animal were analyzed for co-localization with NeuN or GFAP respectively. The percentage of BrdU-positive cells co-labeled with GFAP, with DCX, or with NeuN was calculated.

DCX^+^ and caspase3^+^ cell count was performed using BZ-II analyzer software (Keyence, Japan). For accurate assessment of caspase3^+^ cells, 100x objective was used. Cells were counted in a delineated area in the DG including GCL and SGZ. Every twelfth hippocampal section was used for analysis. The mean cell density in each mouse was expressed as number of cells/mm^2^.

Boundaries of DG were outlined in every sixth Cresyl violet-stained sections, through the whole rostrocaudal extent of the hippocampus were to determine the total volume of the GCL of the DG. The sum of the outlined DG areas per animal was then multiplied by six and section thickness (40μm) to estimate the total volume according to Cavalieri principle [40, 71]. Volumes are shown as mm^3^.

Mean intensity of Sirt1-immunoreaction (Sirt1-Ir) in GCL and SGZ of 3-4 sections per animal was determined. In each slice, the value of the background intensity in areas without positive signal was measured and subtracted from the specific signal. Quantification of fluorescence intensity was performed using Image J software. Mean fluorescence intensity was expressed in arbitrary unites corresponding to the measured grey values. PMP70 immunoreactivity in DG was assessed in every twelfth section. The percentage of positively PMP70-stained area to the total area of DG was assessed in each section using Image J software (http://rsbweb.nih.gov/ij).

### Analysis of relative gene expression

Three to four adult and young mice, from both genotypes, were killed by an overdose of isoflurane. All animals were killed at a consistent time of day (ZT3) to avoid possible circadian variation of gene expression. Brains were removed quickly. Entire hippocampi were collected, frozen in liquid N_2_ and stored at −80° C. Total RNA from the hippocampi was isolated using RNeasy Lipid Tissue Mini Kit (Qiagen, Germany) according to the manufacturer's protocol. cDNA was prepared using QuantiTect Reverse Transcription Kit (Qiagen, Germany). The following PCR program was used for amplification: 15 min at 42° C, 1 min at 95°C then at 4°C. Real time PCR was performed using Applied Biosystems StepOne™ Real-Time PCR Systems and Fast SYBR® Green Master Mix (Applied Biosystems, USA). Primers used are listed in Table 1. Beta actin and ribosomal protein S11 (*rps11)* were used as house-keeping genes. The average of the Ct-values of both housekeeping genes was used to calculate the relative expression levels for the respective genes of interest [72].

**Table 1:** Primer list

	Forward	Reverse
***beta actin***	**CCCAGATCATGTTTGAGACCTT**	**GGTACGACCAGAGGCATACAG**
***bdnf***	**TACCTGGATGCCGCAAACAT**	**GCTGTGACCCACTCGCTAAT**
***fgf***	**CGAGAAGAGCGACCCACAC**	**TGTAACACACTTAGAAGCCAGCA**
***cd1***	**CCCTGGAGCCCTTGAAGAAG**	**AGATGCACAACTTCTCGGCA**
***mt***	**TCCGATGGATCCTGCTCCT**	**AGCAGCAGCTTTTCTTGCAG**
***p21*** ^*Waf1/CIP1*^	**GCAAAGTGTGCCGTTGTCTC**	**CGTCTCCGTGACGAAGTCAA**
***prdx 1***	**TGGCGCTTCTGTGGATTCTC**	**CTGAGCAATGGTGCGCTTG**
***rps11***	**TCGAGGGCACCTACATAGACA**	**GGGGACAGGTGCACAGACAT**
***wee***	**AGAAAGAGCGCAGAGCAGTT**	**TCTGTGAAGAGTGCCCGTTC**

### Statistical analysis

Statistical analysis was performed using Graph Pad Prism software. Mann-Whitney-U test or Student's T-test were used to determine differences between groups. Values are presented as mean ± SEM. *P* value < 0.05 was considered statistically significant.

## Abbreviations

SGZ: subgranular zone
DG: dentate gyrus
SVZ: subventricular zone
BMAL1: brain and muscle Arnt‐like protein1
CLOCK: circadian locomotor output cycles
Bmal1^‐/‐^: Bmal1‐deficient mice
Bmal1^+/+^: wildtype littermates
NPCs: neuronal progenitor/precursor cells
Sirt1: Sirtuine 1
PMP70: Peroxisomal membrane protein at 70kDa
BrdU: bromodeoxyuridine
DAB: diaminobezidine
GCL: granular cell layer
NAD: nicotinamide adenine dinucleotide
HDAC: histone deacetylase
prdx 1: peroxiredoxin 1
mt: metallothionein
FGF: fibroblast growth factor
BDNF: brain derived neurotrophic factor
ROS: reactive oxygen species
